# Mediation analysis of leisure activities on the association between cognitive function and mortality: a longitudinal study of 42,942 Chinese adults 65 years and older

**DOI:** 10.4178/epih.e2022112

**Published:** 2022-11-27

**Authors:** Xingxing Chen, Wenfan Wu, Xian Zhang, Tingxi Long, Wenyu Zhu, Rundong Hu, Xurui Jin, Lijing L. Yan, Yao Yao

**Affiliations:** 1 School of Public Health, Wuhan University, Wuhan, China; 2 Global Health Research Center, Duke Kunshan University, Kunshan, China; 3 MindRank AI Ltd., Hangzhou, China; 4 Department of Geriatrics, Affiliated Kunshan Hospital of Jiangsu University, Kunshan, China; 5 Duke Global Health Institute, Duke University, Durham, NC, USA; 6 Institute for Global Health and Management, Peking University, Beijing, China; 7 China Center for Health Development Studies, Peking University, Beijing, China; 8 Center for Healthy Aging and Development Studies at National School of Development, Peking University, Beijing, China

**Keywords:** Causal mediation analysis, Older people, Cognitive function, All-cause mortality, Leisure activities

## Abstract

**OBJECTIVES:**

Previous studies have established associations of cognitive function and leisure activities with mortality. This study aimed to evaluate whether leisure activities causally mediate these associations.

**METHODS:**

This longitudinal study included 42,246 participants aged over 65 years from the Chinese Longitudinal Healthy Longevity Survey. The Mini-Mental State Examination and a self-reported scale were used to measure cognitive status and leisure activities, respectively. We examined the associations of cognitive function and leisure activities with mortality using Cox proportional hazards models. Causal mediation analysis was used to assess whether leisure activities mediated the association between cognitive function and mortality.

**RESULTS:**

Cognitive function and leisure activities were inversely associated with mortality. Leisure activities accounted for 28.3% (95% confidence interval [CI], 25.6 to 31.1) of the total effect of cognitive function and mortality. A higher mediated proportion (PM) was observed for physical leisure activities (PM, 20.1%; 95% CI, 18.0 to 22.3) than for social leisure activities (PM, 17.7%; 95% CI, 15.7 to 19.7). The mediating effect was higher among participants at younger ages (PM, 41.5%; 95% CI, 21.3 to 65.4), those with higher education levels (PM, 30.5%; 95% CI, 25.3 to 36.2), and residents of rural China (PM, 42.5%; 95% CI, 25.4 to 62.5).

**CONCLUSIONS:**

Cognitive function was associated with inverse mortality. Leisure activities significantly mediated this association. Participation in leisure activities at the early stages of mild cognitive impairment could reduce the risk of mortality, which has a major impact on interventional strategies for healthy aging.

## GRAPHICAL ABSTRACT

[Fig f3-epih-44-e2022112]

## INTRODUCTION

A series of epidemiological studies have demonstrated that lower cognitive function is associated with adverse outcomes such as increased use of home healthcare [1], hospitalization [2], and mortality in older adults [3–6]. However, the mediators of this pathway, through which cognitive impairment increases the mortality rate, remain unclear. The number of people with cognitive impairment is growing with the rapid progress of population aging, resulting in a prioritization of interventions aiming to reduce the risk of mortality due to cognitive impairment.

Previous studies have shown that cognitive impairment increases the risk of developing neurodegenerative diseases (e.g., stroke or dementia), which in turn is related to higher rates of mortality [7]. However, interventions to delay the progress of neurodegenerative diseases have exhibited poorer performance in reducing the mortality rates among people with cognitive impairment [8]. Accordingly, more studies are needed to study the mediators between cognitive impairment and mortality to provide more evidence for designing such interventions. In a recent observational study, leisure activities demonstrated a greater potential to reduce mortality in older adults with cognitive impairment compared to those who were not cognitively impaired [9]. This finding may indicate that leisure activities could serve as a mechanism for explaining the association between cognitive function and mortality. Even so, there is a lack of evidence relating leisure activities to cognitive function and mortality, particularly those exploring their causal relationship.

It is difficult to determine whether leisure activities mediate the relationship between cognitive function and mortality statistically. Although the commonly used structural equation modeling method can explore the mediating path by using both cross-sectional data and longitudinal data, it performs poorly when handling survival data. A newly developed longitudinal causal mediation approach may provide a solution [10]. This analysis is based on a counterfactual framework [11], which could be used to clarify the causality between cognitive function and mortality, and to identify potential mediating pathways that provide evidence for interventional study designs.

We aimed to explore the causal mediating effect of leisure activities on the association between cognitive function and mortality among a nationwide population-based sample of Chinese adults aged 65 years or older. We hypothesized that leisure activities would mediate the association between cognitive function and mortality.

## MATERIALS AND METHODS

### Study design and participants

We used data from the Chinese Longitudinal Healthy Longevity Survey (CLHLS), which has been conducted since 1998 with follow-ups every 2–3 years. The CLHLS surveys were conducted in randomly selected counties and cities that accounted for half of all counties and cities in 23 out of the 31 provinces of China, covering over 85% of China’s population. The CLHLS sampling frame started with lists of centenarians that are available at the county/city level (lists of adults aged 80+ are not readily available at this level because their population size is too large). Almost all centenarians were interviewed, with few refusals. For every centenarian, the CLHLS interviewed a randomly selected nearby octogenarian and a nearby nonagenarian with a predesignated age and sex. The term “nearby” refers to the same village or street, or the same town, county, or city, where applicable. The goal of the sampling strategy was to include comparable numbers of randomly selected male and female at ages 80 to 99 so that each subsequent wave could have a sufficient number of very old respondents in the sample. Starting in the third wave in 2002, the CLHLS included young-old adults aged 65 to 79 as a comparison group. The sample selection strategy for the young-old adults was the same as that for the oldest-old. As all adults aged 65 to 99 were randomly selected, the CLHLS sample represents older adults in China very well. All data were collected through face-to-face interviews by trained interviewers who were local staff members from the county-level network system of the National Bureau of Statistics of China.

We pooled 43,487 participants aged 65 years or above at baseline from the 1998 to 2011/2012 waves, with the latest follow-up until 2014 from the CLHLS. To select a group meeting the criteria, we excluded 57 respondents whose age was below 65 and 488 respondents lost to follow-up in the 2014 wave. We also dropped 696 cases with missing information on key variables, leaving a sample of 42,246 for the analysis, which included 16,445 survivors and 25,801 deaths. The details of this survey have been published elsewhere [12]. The follow-up period was up to 16.5 years (median, 3.78), and 61% (n=25,801) of participants died during the follow-up period until 2014. The flow chart of study population is shown in Supplementary Material 1.

### Exposure

The exposure was cognitive function, which was assessed for all participants using the Chinese version of the Mini-Mental State Examination (MMSE) during each survey through a home-based interview.

**Figure 1:**
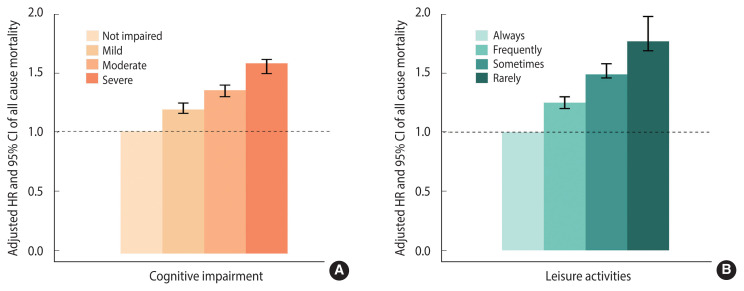
Multivariable-adjusted hazard ratios (HRs) and 95% confidence intervals (CIs) of mortality by cognitive impairment (A) and leisure activities (B) among Chinese adults aged ≥65 years (adjusted for age, sex, residence, smoking status, drinking status, tea drinking, regular physical activity, lifestyle, and eight kinds of self-reported disease: heart disease, stroke, diabetes, cancer, chronic obstructive pulmonary disease, hypertension, Parkinson’s disease, and bedsore).

The Chinese MMSE has been proven to be reliable and valid in previous studies [13–15]. The Chinese MMSE consists of 24 components encompassing 7 subdomains: orientation (4 components for time orientation and 1 component for place orientation); naming foods (naming as many kinds of food as possible in 1 minute, one component); registration of 3 words 3 components; attention and calculation (mentally subtracting 3 iteratively from 20, 5 components); copying a figure (1 component); recall (delayed recall of the 3 words mentioned above, 3 components); and language (2 components for naming objectives, 1 component for repeating a sentence, and 3 components for listening and obeying).

According to the literature [16,17], we classified cognitive impairment into 4 groups (not impaired: 30–25 [MMSE score]; mild: 24–18; moderate: 17–10; severe: 9–0). In our study, we considered responses of “unable to answer” as a “wrong answer” [18,19]. The MMSE score ranged from 0 to 30, with a lower score indicating worse cognitive function.

### Mediator

Data on self-reported leisure activities were collected through 8 questions to assess the type of leisure activities in which respondents most frequently participated.

Respondents were asked if they were presently engaged in 8 leisure components, including: (1) housework (cooking or childcare), (2) fieldwork, (3) garden work, (4) reading newspapers/books, (5) raising domestic animals, (6) playing cards and/or mahjong, (7) watching television and/or listening to the radio, and (8) social activities. We then grouped the 8 leisure activities into 2 categories: social leisure activities (housework, playing cards/mahjongg, and social activities), and physical leisure activities (doing fieldwork, gardenwork, reading newspapers/books, watching television/listening to the radio, and raising domestic animals) [20].

For each leisure component, the frequency was categorized into 3 scores: “almost every day” (scored as 3 points), “sometimes or occasionally” (scored as 2 points), and “rarely or never” (scored as 1 point). Composite scores were calculated for social leisure activities (3 items, ranging from 3 to 9), physical leisure activities (5 items, ranging from 5 to 15), and leisure activities (the sum of all 8 activities, ranging from 8 to 24) separately [21]. The reliability and validity of self-reported leisure activities have been examined in previous research [20].

### Outcome

Information on vital status and date of death were collected from officially issued death certificates provided by the local government department. When such information was not available, knowledgeable relatives of the decedents were interviewed. The duration of follow-up was calculated by the time interval between the first interview date and the date of death. Participants who survived the last interview were regarded as being censored on the date of their last interview in 2014.

### Covariates

All covariates included in the present study were categorized into the following 3 groups: demographic characteristics, health behaviors, and disease conditions. Demographic characteristics included age at baseline, sex, education attainment (none, primary school, or middle school or higher), ethnicity (Han vs. minority), place of residence (urban vs. rural), marital status (currently married and living with their spouse, separated/divorced/never married, or widowed), and occupation before 60 years old (manual vs. non-manual). Health behaviors included smoking status (never vs. ever), drinking status (never vs. ever), frequency of drinking tea (every day or occasionally vs. rarely or never), lifestyle (healthy vs. unhealthy) and regular physical activity (yes vs. no). Details on disease conditions were collected through a series of self-reported questions on diseases. The following 8 kinds of self-reported diseases (dichotomized as with vs. without) were included in our study: heart disease, stroke, diabetes, cancer, chronic obstructive pulmonary disease, hypertension, Parkinson’s disease, and bedsore.

### Statistical analysis

We described the baseline characteristics of the study sample, providing the count and percentage for categorical variables and median and interquartile range for continuous variables. The Kruskal-Wallis H-test for continuous variables and the chi-square test for categorical variables were applied to compare differences by different cognitive function categories. Hazard ratios (HRs) and 95% confidence intervals (CIs) were used to determine the associations between cognitive function, leisure activities, and mortality using Cox proportional-hazard regression models. We tested and ascertained that the proportional-hazard assumption had not been violated.

A simple conceptual diagram of the current study is shown in Supplementary Material 2. Considering the cognition-related components contained in leisure activities and their features in the causal pathway between cognitive function and mortality, any adjustment for leisure activities as a confounder would render the associations biased [22]. Thus, we undertook a causal mediation analysis based on a counterfactual framework to assess the mediating role of leisure activities in the association between cognitive function and mortality using 3-way decomposition [23,24], which decomposes the total effect (TE) into 3 components. The definitions and interpretations of each of these components in the context of this study are shown in Table 1.

In order to assess disparities across different populations, we also conducted subgroup analyses by age groups (65–79 vs. ≥80 years), education (no schooling vs. primary or higher), lifestyle (healthy vs. unhealthy), and residence (urban vs. rural), respectively. We also conducted causal mediation analyses for 7 subdomains of cognitive function and mortality.

We conducted several sensitivity analyses to check the robustness of the results: (1) including participants who had missing values for key variables to examine whether the missing data affected the robustness of the results; (2) excluding participants who died within 2 years after the baseline survey when survival analysis was applied; (3) applying an interaction effect when conducting causal mediation analysis; (4) estimating the controlled direct effect (CDE) across the range of leisure activities score from 8 to 24 to observe a moderating effect.

The 3-way decomposition was conducted using R (‘*regmedint*’) [25]. A 2-tailed p-value less than 0.05 was considered statistically significant. All analyses were performed using R version 4.1.1 (R Core Team, Vienna, Austria).

### Ethics statement

The CLHLS was approved by the Biomedical Ethics Committee of Peking University (IRB00001052-13074) and was conducted in accordance with the guidance of the Declaration of Helsinki.

## RESULTS

### Baseline characteristics

Table 2 presents the descriptive baseline characteristics of different cognitive function groups. The mean age of participants was 91 years (range, 65–122) and more than half of them (59.1%) were female. The self-reported mean score of leisure activities was 12 (25th and 75th quantiles: 9 and 14). Participants with worse cognitive function (i.e., lower scores on the MMSE) were more likely to be older, female, lower educated, and ethnic Han, to live in rural areas, to be widowed or separated, and to have had manual occupations, lack physical activity, frequently consume tea, have an unhealthy lifestyle, and have lower leisure activity scores (all p<0.001). Participants with worse cognitive function were less likely to smoke and drink (all p<0.001).

### Cognitive function, leisure activities, and mortality

The multivariate Cox models demonstrated dose–response patterns between cognitive impairment and mortality (Figure 1A). A lower MMSE score was associated with a higher rate of mortality (reference: not impaired, mild: HR, 1.17; 95% CI, 1.14 to 1.22, moderate: HR, 1.32; 95% CI, 1.27 to 1.36, severe: HR, 1.53; 95% CI, 1.45 to 1.56). Leisure activities and mortality showed a consistent dose-response relationship (Figure 1B). Compared with those who participated in leisure activities regularly, those who seldom did so had an increased risk of mortality, with a HR of 1.77 (95% CI, 1.69 to 1.98).

### Causal mediation analysis

**Figure 2:**
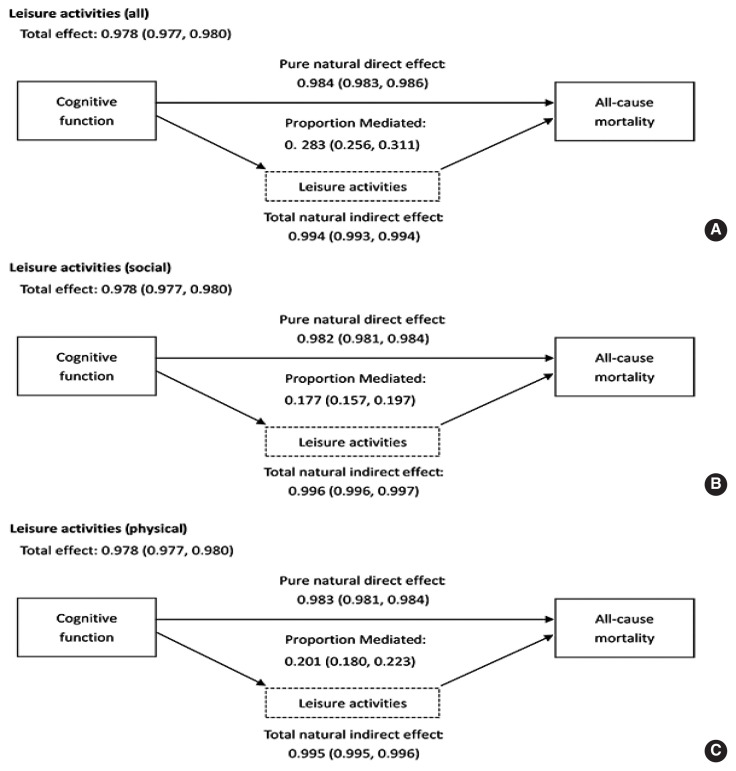
Causal mediating effect of leisure activities (A: all, B: Social, and C: physical) on the association between cognitive function and all-cause mortality (adjusted for age, sex, residence, smoking status, drinking status, tea drinking, regular physical activity, lifestyle, and eight kinds of self-reported disease). Values are presented as adjusted hazard ratio (95% confidence interval).

The results of the causal mediation analysis are shown in Figure 2. The TE between cognitive function and mortality had an HR of 0.978 (95% CI, 0.977 to 0.980). The HRs for the pure natural direct effect (PNDE) (direct effect of cognitive function on mortality) and the total natural indirect effect (TNIE) (mediated effect of leisure activities) were 0.984 (95% CI, 0.983 to 0.986) and 0.994 (95% CI, 0.993 to 0.994), respectively. Thus, 28.3% (95% CI, 25.6 to 31.1) of the TE of cognitive function and mortality was associated with leisure activities. A higher mediated proportion (PM) was observed for physical leisure activities (PM, 20.1%; 95% CI, 18.0 to 22.3) than for social leisure activities (PM, 17.7%; 95% CI, 15.7 to 19.7).

### Subgroup analysis

The causal mediating effect of leisure activities was higher among participants at younger ages (PM, 41.5%; 95% CI, 21.3 to 65.4), those with higher education levels (PM, 30.5%; 95% CI, 25.3 to 36.2), those with an unhealthy lifestyle (PM, 32.2%; 95% CI, 20.5 to 43.6), and residents of rural China (PM, 42.5%; 95% CI, 25.4 to 62.5) (Table 3 and Supplementary Material 3).

The results of further causal mediation analysis showed that leisure activities causally mediated the associations between 7 subdomains of cognitive function and mortality (all p<0.001) (Supplementary Material 4).

### Sensitivity analysis

The TE was robust when applying the interaction effect (Supplementary Material 5), while PNDE, TNIE, and PM showed similar patterns to TE, proving the robustness of 3-way decomposition for the causal mediation estimate. For CDE, the results suggested no moderation effect by fixing the leisure activities score in the range of 8–24 (all HR, 0.984, all p<0.001). We included participants who had missing values for key variables and then carried out the causal mediation analysis, and the results showed that the imputed data would not cause bias when estimating the effect (Supplementary Material 6). After excluding participants who died within 2 years after baseline (Supplementary Material 7), the dose-response relationships of both cognitive function and leisure activities with mortality remained consistent.

## DISCUSSION

We found that cognitive function and leisure activities were negatively associated with mortality in this prospective cohort study of 42,942 Chinese older adults. In the causal mediation analysis, leisure activities were an important mediator of the association between cognitive function and mortality, with indirect effects via leisure activities contributing 28.3% of the TE. Engaging in leisure activities at an early stage of mild cognitive impairment may reduce the risk of mortality.

This study confirmed the dose-response relationships of cognitive function and leisure activities with mortality in Chinese older adults, in line with another report [26]. Few studies have examined the relationship between cognitive function and mortality in China to date [27,28], but accumulating evidence supports our conclusion that cognitive function is an independent predictor of an increased risk of death in older people [13]. Furthermore, we also provide more evidence on the association between leisure activities and mortality among Chinese older adults, including watching TV or listening to the radio, playing cards or mahjong, reading newspapers or books, keeping domestic animals or pets, and attending social activities. Our finding is in accordance with a previous study showing that older adults who engaged in a greater number of activities were less likely to die than were those who engaged in few activities [29]. Thus, encouraging older adults to participate in these activities can help reduce the risk of death.

This study also provides evidence that contributes to explaining the mechanisms underlying the association between cognitive function and mortality and supports the view that leisure activities play an important role in this association. People with impaired cognitive function commonly have a combination of social isolation and depression [30,31], with which a loss of participation in social or physical leisure activities would occur after an early stage of cognitive deficit has developed owing to preclinical dementia. Discontinuing engagement in leisure activities may constitute an early sign of dementia incidence [32], and such potential dementia cases are more likely to experience cognitive decline eventually, resulting in higher rates of mortality. Thus, it’s a priority to reduce the risk of cognition-related mortality. It has been found that engaging in leisure activities could increase the resilience against the negative effects of cognitive impairment among those older adults and further reduce the mortality risk coming from cognitive impairment [27,33]. Additionally, previous studies have also put forward to 2 principles to guide engagement in leisure activities, that is, engaging in complex leisure activities and enabling people to engage in complex leisure activities at an early stage of cognitive impairment [34]. The effects of implementing these 2 principles for leisure activities have been confirmed in older people and patients with dementia [35]. Although the principles were developed on the basis of findings from Western countries, they are consistent with our results from older Chinses adults. Leisure activities are not a static property and they could be the target for intervention; improving leisure activities may contribute to protecting older people against cognitive decline and reducing the mortality risk that comes from cognitive impairment.

We also evaluated the distinct causal effects of different types of leisure activities on the association between cognitive function and mortality. A previous study with 10,308 participants in the United Kingdom explicated that social leisure activities had a stronger and more consistent relationship with cognitive function and mortality than physical leisure activities [34]. The mechanisms may include support (received or provided), reduced depression, and positive emotions leading to the health-promoting physiological effects of decreased chronic sympatho-adrenal activation, improved immune function, and less chronic inflammation [36–38]. However, our results indicated that the PM for physical leisure activities was higher than that of social leisure activities. A plausible explanation for this finding is that physical leisure activities could increase the high-density lipoprotein level, reduce coronary artery calcium, and improve antioxidant defense systems [39]. It is also possibly explained by the different characteristics of older people between China and other countries. Combined with previous evidence, these results suggest that planned interventions for leisure activities, especially physical activities, at an early stage of mild cognitive impairment could promote health outcomes and optimally reduce the risk of mortality.

Our results also suggest that leisure activities have a causal mediating effect on the association between cognitive function and mortality in different subgroups of age, educational attainment, lifestyle, and residence. We found that the PM of leisure activities in rural China was much higher than that in urban. Given the huge urban-rural differences in China, rural dwellers had fewer opportunities for social interactions, recreational facilities, and sports that are easily available to urban dwellers, resulting in poor cognitive performance and a higher risk of mortality [40], as well as a higher proportion mediated by leisure activities. Although these results reflect the relatively low availability of leisure resources and fewer choices for alternative activities in rural China, the findings from this study suggest that engagement in leisure activities might be more cost-effective for minimizing the risks of mortality associated with cognitive function in rural areas, in light of the relatively low degrees of leisure activities in those regions. Compared with the oldest old, the mediated magnitude for leisure activities was higher in younger-old adults in the present study. The results of our study have a similar pattern to previous work, according to which younger-old adults may develop cognitive impairment faster than the oldest-old [41]. Furthermore, it was observed that the association of cognitive impairment with mortality decreased with age in older Chinese cohorts [42]. Thus, whenever possible, early engagement in leisure activities for cognitively impaired younger-old adults may have a large impact on mortality in later life. Previously, education was found to play a moderating role in the relationship between cognitive performance and leisure activities in older adults [43]. Moreover, the relationship between cognitive function and leisure activities differed depending on the educational level; that is, the impacts of leisure activities on cognition were more profound for educated older people [22]. Our findings are consistent with those results that participating in leisure activities is more likely to reduce the risk of mortality for higher-educated older people. In addition, it is reasonable that the PM was higher in older adults with an unhealthy lifestyle than those with a healthy lifestyle, as older adults with unhealthy lifestyles are more likely to benefit from other healthy behaviors such as leisure activities [44].

The results of this study have important implications for public health. Interventions at an early stage of cognitive impairment have been shown to be most effective, and even small effects may lead to significant public health benefits [45]. Many factors that contribute to the association between cognitive decline and mortality, such as advanced age and neurodegenerative diseases, are not modifiable. However, older people can readily modify their behaviors. By encouraging older people to participate in leisure activities, we can help to weaken the association between cognitive impairment and death. Based on longitudinal data, this study is the first of its kind to examine the mediating role of leisure activities on the association between cognitive function and mortality. A large sample size allowed us to adjust for a large number of covariates and conduct detailed analyses.

Nonetheless, there are several limitations to this study. First, the MMSE is a brief measure of global cognitive function, which might not be sensitive enough. Second, we used the first interview date as a proxy for the date of diagnosis of cognitive impairment. Some patients may have been diagnosed a long time before our first interview, which may have affected their survival time, potentially biasing the results. Last, we did not exclude participants with cognitive impairment, which may increase the risk of reverse causality, as people in the very early stages of cognitive impairment may withdraw from social contact or other types of leisure activities. However, the use of longitudinal follow-up data and causal mediation models could counteract this risk to a certain extent.

The present study showed a dose-response relationship between cognitive function and mortality. Leisure activities mediated this relationship. Early intervention for mild cognitive impairment, focusing mostly on physical leisure activities, could reduce the risk of mortality related to cognition.

## Figures and Tables

**Figure f3-epih-44-e2022112:**
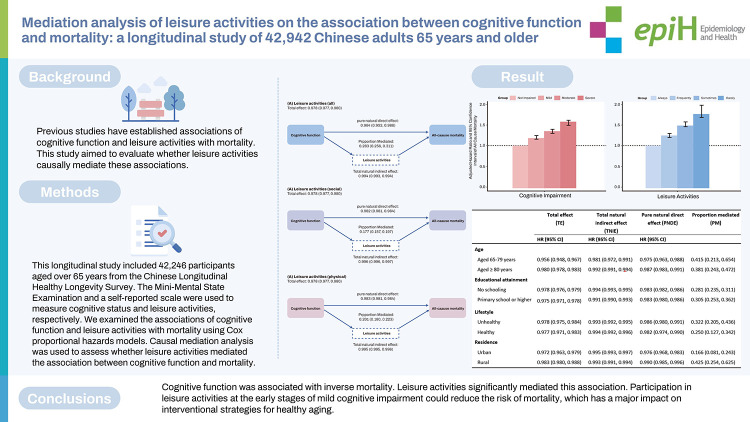


**Table 1.** Definitions of the components of the 3-way decomposition in this study^1^

Components	Counterfactual definition	Explanation	Contextual definition
Total effect	*Y*_1_−*Y*_0_	Total effect of exposure *A* (changing from a_0_ to a_1_) on the outcome Y	By how much time is the survival time prolonged or shortened, when the cognitive function score increases or decreases by 1 unit?
Total natural indirect effect	*Y*_1*M*1_−*Y*_1*M*0_	The effect of allowing each individual’s mediator to change from its natural value had the individual received exposure level a_0_ to its natural value had the individual received exposure level a_1_, while holding constant the exposure *A* to level a_1_; Since the mediator is allowed to change, this is the part of the exposure effect exerted through its impact on the mediator	By how much time is the survival time prolonged or shortened, when the cognitive function score increases or decreases by 1 unit, if cognitive function had an effect on leisure activities?
Pure natural direct effect	*Y*_1*M*0_−*Y*_0*M*0_	The effect of changing exposure *A* from a_0_ to a_1_ while the mediator is held at each individual’s natural value taken under exposure level a_0_; Since the mediator is not allowed to change, this effect is outside the pathway through the mediator	By how much time is the survival time prolonged or shortened, when only considering the increase or decrease on cognitive function?
Controlled direct effect	*Y*_1*M*1_−*Y*_0*M*1_	The effect of exposure *A* (changing from a_0_ to a_1_) on the outcome *Y*, with the mediator *M* fixed at level *M*_1_; The controlled direct effect defines the component of the total effect that is due neither to interaction nor mediation	By how much time is the survival time prolonged or shortened, when the cognitive function score increases or decreases by 1 unit, if everyone attained the same given frequency of leisure activities?
Proportion mediated	(*Y*_11_−*Y*_10_−*Y*_01_−*Y*_00_) (*M*_1_−*M*_0_)	The proportion of the effect of changing exposure *A* from a_0_ to a_1_ on the outcome *Y* that occurs via influencing the mediator	The proportion of the effect of cognitive function on all-cause mortality explained by leisure activities to the total effect

*M*, mediator (leisure activities); *A*(a), exposure (cognitive function); *Y*, outcome (all-cause mortality).

1 a_1_ and a_0_ do not represent the presence or absence of cognitive impairment, but the change of cognitive function from one level to another; *M*_1_ and *M*_0_ are similar to this.

**Table 2.** Baseline characteristics by different cognitive function groups among Chinese adults aged ≥65 years (n=42,246)

Characteristics		Cognitive impairment^1^					
		Not impaired	Mild	Moderate	Severe	Total	p-value
Total		22,216 (52.6)	8,855 (21.0)	4,651 (11.0)	6,524 (15.4)	42,246 (100)	
Age, median (25th, 75th) (yr)		83 (75, 91)	92 (85, 100)	97 (91, 101)	100 (94, 101)	91 (80, 99)	<0.001
Sex							<0.001
	Male	11,705 (52.7)	2,948 (33.3)	1,148 (24.7)	1,465 (22.5)	17,266 (40.9)	
	Female	10,511 (47.3)	5,907 (66.7)	3,503 (75.3)	5,059 (77.5)	24,980 (59.1)	
Educational attainment^2^							<0.001
	No schooling	11,338 (51.0)	6,818 (77.0)	3,974 (85.5)	5,536 (84.8)	27,666 (65.5)	
	Primary school	6,263 (28.2)	1,475 (16.7)	513 (11.0)	702 (10.8)	8,953 (21.2)	
	Middle school or higher	4,615 (20.8)	562 (6.3)	164 (3.5)	286 (4.4)	5,627 (13.3)	
Main occupation before age 60							<0.001
	Non-manual	2,427 (10.9)	254 (2.9)	71 (1.5)	143 (2.2)	2,895 (6.9)	
	Manual	19,789 (89.1)	8,601 (97.1)	4,580 (98.5)	6,381 (97.8)	39,351 (93.1)	
Ethnicity							<0.001
	Han	20,860 (93.9)	8,318 (93.9)	4,389 (94.4)	6,217 (95.3)	39,784 (94.2)	
	Others (minority)	1,356 (6.1)	537 (6.1)	262 (5.6)	307 (4.7)	2,462 (5.8)	
Residence							<0.001
	Urban	10,041 (45.2)	3,486 (39.4)	1,689 (36.3)	2,483 (38.1)	17,699 (41.9)	
	Rural	12,175 (54.8)	5,369(60.6)	2,962 (63.7)	4,041(61.9)	24,547 (58.1)	
Marital status							<0.001
	Currently married and living with spouse	8,555 (38.5)	1,470 (16.6)	373 (8.0)	437 (6.7)	10,835 (25.6)	
	Others^3^	13,661 (61.5)	7,385 (83.4)	4,278 (92.0)	6,087 (93.3)	31,411 (74.4)	
Tobacco smoking status							<0.001
	Never	13,353 (60.1)	6,469 (73.1)	3,652 (78.5)	5,227 (80.1)	28,701 (67.9)	
	Ever	8,863 (39.9)	2,386 (26.9)	999 (21.5)	1,297 (19.9)	13,545 (32.1)	
Alcohol drinking status							<0.001
	Never	14,676 (66.1)	6,361 (71.8)	3,548 (76.3)	4,978 (76.3)	29,563 (70.0)	
	Ever	7,540 (33.9)	2,494 (28.2)	1,103 (23.7)	1,546 (23.7)	12,683 (30.0)	
Regular physical activity							<0.001
	Yes	9,708 (43.7)	2,754 (31.1)	1,093 (23.5)	1,448 (22.2)	15,003 (35.5)	
	No	12,508 (56.3)	6,101 (68.9)	3,558 (76.5)	5,076 (77.8)	27,243 (64.5)	
Frequency of drinking tea							<0.001
	Every day or occasionally	11,419 (51.4)	3,781 (42.7)	1,725 (37.1)	1,990 (30.5)	18,915 (44.8)	
	Rarely or never	10,797 (48.6)	5,074 (57.3)	2,926 (62.9)	4,534 (69.5)	23,331 (55.2)	
Lifestyle^4^							<0.001
	Unhealthy	10,930 (49.2)	5,588 (63.1)	3,195 (68.7)	4,502 (69.0)	24,215 (57.3)	
	Healthy	11,286 (50.8)	3,267 (36.9)	1,456 (31.3)	2,022 (31.0)	18,031 (42.7)	
Leisure activities, median (25th, 75th)^5^		13 (11, 15)	11 (9, 13)	10 (8, 11)	9 (8, 10)	12 (9, 14)	<0.001

Values are presented as number (%). MMSE, Mini-Mental State Examination.

1 Cognitive impairment was classified into 4 mutually exclusive groups: not impaired (25≤MMSE score≤30), mild (18≤MMSE score≤24), moderate (10≤MMSE score≤17), and severe (0≤MMSE score≤9) cognitive impairment.

2 Defined by years of schooling; None: school years=0; Primary school: school years=1-5; Middle school or higher: school years>5.

3 ‘Others’ include widowed, separated, divorced and never married.

4 The lifestyle score was calculated by the intake frequency of 10 food categories that have been demonstrated to be associated with cognitive function; A 3-point scale question was used to measure the current intake frequency of each food group: “always or almost every day,” “sometimes or occasionally,” or “rarely or never”; Those 3 terms received a score of 2, 1, or 0, respectively; The total score ranged from 0 to 20, and lifestyle was categorized as unhealthy or healthy using a cut-off score of 10.

5 The leisure activity score was calculated by 8 kinds of activities (housework, fieldwork, garden work, reading, pets, mahjong, television, social-activity) and we scored each activity 1 for “never,” 2 for “sometimes,” and 3 for “almost every day”; The score ranged from 6 to 24, with higher scores indicating more frequent leisure activities.

**Table 3.** Causal mediation analysis of leisure activities on the association between cognitive function and all-cause mortality in subgroups among Chinese adults aged ≥65 years^1^

Variables		Total effect	p-value	Total natural indirect effect	p-value	Pure natural direct effect	p-value	Proportion mediated	p-value
Age (yr)									
	65-79	0.956 (0.948, 0.967)	<0.001	0.981 (0.972, 0.991)	<0.001	0.975 (0.963, 0.988)	<0.001	0.415 (0.213, 0.654)	<0.001
	≥80	0.980 (0.978, 0.983)	<0.001	0.992 (0.991, 0.994)	<0.001	0.987 (0.983, 0.991)	<0.001	0.381 (0.243, 0.472)	<0.001
Educational attainment									
	No schooling	0.978 (0.976, 0.979)	<0.001	0.994 (0.993, 0.995)	<0.001	0.983 (0.982, 0.986)	<0.001	0.281 (0.235, 0.311)	<0.001
	Primary school or higher	0.975 (0.971, 0.978)	<0.001	0.991 (0.990, 0.993)	<0.001	0.983 (0.980, 0.986)	0.002	0.305 (0.253, 0.362)	<0.001
Lifestyle									
	Unhealthy	0.978 (0.975, 0.984)	<0.001	0.993 (0.992, 0.995)	<0.001	0.986 (0.980, 0.991)	<0.001	0.322 (0.205, 0.436)	<0.001
	Healthy	0.977 (0.971, 0.983)	<0.001	0.994 (0.992, 0.996)	<0.001	0.982 (0.974, 0.990)	<0.001	0.250 (0.127, 0.342)	<0.001
Residence									
	Urban	0.972 (0.963, 0.979)	<0.001	0.995 (0.993, 0.997)	<0.001	0.976 (0.968, 0.983)	<0.001	0.166 (0.081, 0.243)	<0.001
	Rural	0.983 (0.980, 0.988)	<0.001	0.993 (0.991, 0.994)	<0.001	0.990 (0.985, 0.996)	<0.001	0.425 (0.254, 0.625)	<0.001

Values are presented as hazard ratio (95% confidence interval).

1 Adjusted for age, sex, residence, smoking status, drinking status, tea drinking, regular physical activity, lifestyle, and 8 kinds of self-reported diseases.
