# Supplementary material for: Mediation analysis of leisure activities on the association between cognitive function and mortality: a longitudinal study of 42,942 Chinese adults 65 years and older
Source: Epidemiol Health. 2022 Nov 27;44:e2022112. doi: 10.4178/epih.e2022112 (PMC10106552; doi:10.4178/epih.e2022112)
Supplement: Supplementary file 1 [file epih-44-e2022112-Supplementary-1.docx]

**Mediation analysis of leisure activities on the association between cognitive function and mortality: Longitudinal study of 42,942 Chinese adults 65 years and older**

***Supplementary data***

**Supplementary Material 1.** Flow chart of the study population
