# Supplementary material for: Mediation analysis of leisure activities on the association between cognitive function and mortality: a longitudinal study of 42,942 Chinese adults 65 years and older
Source: Epidemiol Health. 2022 Nov 27;44:e2022112. doi: 10.4178/epih.e2022112 (PMC10106552; doi:10.4178/epih.e2022112)
Supplement: Supplementary file 2 [file epih-44-e2022112-Supplementary-2.docx]

Considering the cognition-related components containing in leisure activities and their features on the causal pathway between cognitive function and mortality, such that any adjustment for leisure activities as a confounder will render the associations biased (2) (**Fig. S2**). Does the risk of mortality in relation to cognitive function operate through leisure activities, and if so, by how much? Causal mediation analysis seeks to provide answers to these questions.

**Supplementary Material 2.** Simplified directed acyclic graph depicting the relation between cognitive function and all-cause mortality with leisure activities as the mediator.
