# Supplementary material for: Mediation analysis of leisure activities on the association between cognitive function and mortality: a longitudinal study of 42,942 Chinese adults 65 years and older
Source: Epidemiol Health. 2022 Nov 27;44:e2022112. doi: 10.4178/epih.e2022112 (PMC10106552; doi:10.4178/epih.e2022112)
Supplement: Supplementary file 3 [file epih-44-e2022112-Supplementary-3.docx]

**Supplementary Material 3.** Causal mediation analysis of leisure activities subgroups on the association between cognitive function and all-cause mortality in subgroups among Chinese adults aged ≥ 65 years

|  | **TE** | | **TNIE** | | **PNDE** | | **PM** | |
| --- | --- | --- | --- | --- | --- | --- | --- | --- |
|  | **HR (95% CI)** | ***P* value** | **HR (95% CI)** | ***P* value** | **HR (95% CI)** | ***P* value** |  | ***P* value** |
| **Age** |  |  |  |  |  |  |  |  |
| **Aged 65-79 years** |  |  |  |  |  |  |  |  |
| Leisure activities (social) | 0.957 (0.947, 0.967) | <0.001 | 0.988 (0.980, 0.996) | 0.004 | 0.967 (0.959, 0.979) | <0.001 | 0.272 (0.096, 0.428) | 0.001 |
| Leisure activities (physical) | 0.956 (0.945, 0.966) | <0.001 | 0.986 (0.978, 0.996) | <0.001 | 0.972 (0.960, 0.983) | <0.001 | 0.311 (0.133, 0.509) | 0.001 |
| **Aged ≥ 80 years** |  |  |  |  |  |  |  |  |
| Leisure activities (social) | 0.979 (0.975, 0.982) | <0.001 | 0.996 (0.995, 0.997) | <0.001 | 0.983 (0.978, 0.987) | <0.001 | 0.181 (0.122, 0.249) | <0.001 |
| Leisure activities (physical) | 0.979 (0.977, 0.981) | <0.001 | 0.994 (0.993, 0.995) | <0.001 | 0.985 (0.982, 0.988) | <0.001 | 0.264 (0.219, 0.324) | <0.001 |
| **Education attainment** |  |  |  |  |  |  |  |  |
| **No schooling** |  |  |  |  |  |  |  |  |
| Leisure activities (social) | 0.977 (0.973, 0.981) | <0.001 | 0.996 (0.995, 0.997) | <0.001 | 0.981 (0.975, 0.985) | <0.001 | 0.154 (0.113, 0.216) | <0.001 |
| Leisure activities (physical) | 0.980 (0.975, 0.983) | <0.001 | 0.995 (0.994, 0.996) | <0.001 | 0.985 (0.981, 0.989) | <0.001 | 0.230 (0.156, 0.311) | <0.001 |
| **Primary school or higher** |  |  |  |  |  |  |  |  |
| Leisure activities (social) | 0.974 (0.972, 0.981) | <0.001 | 0.996 (0.995, 0.997) | 0.004 | 0.979 (0.976, 0.984) | <0.001 | 0.154 (0.121, 0.189) | <0.001 |
| Leisure activities (physical) | 0.975 (0.972, 0.978) | <0.001 | 0.994 (0.993, 0.996) | <0.001 | 0.980 (0.978, 0.986) | <0.001 | 0.240 (0.213, 0.284) | <0.001 |
| **Lifestyle** |  |  |  |  |  |  |  |  |
| **Unhealthy** |  |  |  |  |  |  |  |  |
| Leisure activities (social) | 0.975 (0.972, 0.980) | <0.001 | 0.996 (0.995, 0.997) | <0.001 | 0.980 (0.975, 0.985) | <0.001 | 0.152 (0.119, 0.221) | <0.001 |
| Leisure activities (physical) | 0.980 (0.976, 0.984) | <0.001 | 0.995 (0.994, 0.996) | <0.001 | 0.985 (0.981, 0.990) | <0.001 | 0.243 (0.147, 0.341) | <0.001 |
| **Healthy** |  |  |  |  |  |  |  |  |
| Leisure activities (social) | 0.977 (0.971, 0.983) | <0.001 | 0.997 (0.996, 0.998) | <0.001 | 0.979 (0.972, 0.987) | <0.001 | 0.134 (0.044, 0.230) | 0.008 |
| Leisure activities (physical) | 0.978 (0.974, 0.983) | <0.001 | 0.996 (0.994, 0.997) | <0.001 | 0.983 (0.976, 0.988) | <0.001 | 0.209 (0.105, 0.312) | <0.001 |
| **Residence** |  |  |  |  |  |  |  |  |
| **Urban** |  |  |  |  |  |  |  |  |
| Leisure activities (social) | 0.967 (0.961, 0.976) | <0.001 | 0.998 (0.997, 0.999) | 0.005 | 0.971 (0.962, 0.98) | <0.001 | 0.066 (0.010, 0.114) | 0.011 |
| Leisure activities (physical) | 0.975 (0.970, 0.979) | <0.001 | 0.996 (0.994, 0.997) | <0.001 | 0.996(0.995, 0.998) | <0.001 | 0.147 (0.071, 0.233) | <0.001 |
| **Rural** |  |  |  |  |  |  |  |  |
| Leisure activities (social) | 0.978 (0.970, 0.981) | <0.001 | 0.995 (0.992, 0.996) | <0.001 | 0.981 (0.978, 0.985) | <0.001 | 0.188 (0.111, 0.263) | <0.001 |
| Leisure activities (physical) | 0.974 (0.980, 0.988) | <0.001 | 0.995 (0.993, 0.996) | <0.001 | 0.989 (0.985, 0.994) | <0.001 | 0.332 (0.201, 0.469) | <0.001 |

† HR: hazard ratio; CI: confidence interval.

†Adjusted for age, sex, residence, smoking status, drinking status, tea drinking, regular physical activity, lifestyle, and eight kinds of self-reported disease.
