# Supplementary material for: Mediation analysis of leisure activities on the association between cognitive function and mortality: a longitudinal study of 42,942 Chinese adults 65 years and older
Source: Epidemiol Health. 2022 Nov 27;44:e2022112. doi: 10.4178/epih.e2022112 (PMC10106552; doi:10.4178/epih.e2022112)
Supplement: Supplementary file 4 [file epih-44-e2022112-Supplementary-4.docx]

**Supplementary Material 4.** Causal mediation analysis of the effects of leisure activities on the association of seven subdomains of cognitive function and all-cause mortality.

|  | **TE** | | **PNDE** | | **TNIE** | | **PM** | |
| --- | --- | --- | --- | --- | --- | --- | --- | --- |
|  | **HR (95% CI)** | ***P* value** | **HR (95% CI)** | ***P* value** | **HR (95% CI)** | ***P* value** |  | ***P* value** |
| Orientation | 0.911 (0.904, 0.918) | <0.001 | 0.935 (0.928, 0.942) | <0.001 | 0.974 (0.972, 0.976) | <0.001 | 0.277 (0.248, 0.310) | <0.001 |
| Naming food | 0.941 (0.936, 0.946) | <0.001 | 0.958 (0.953, 0.963) | <0.001 | 0.982 (0.981, 0.984) | <0.001 | 0.291 (0.261, 0.322) | <0.001 |
| Registration | 0.892 (0.882, 0.902) | <0.001 | 0.928 (0.918, 0.939) | <0.001 | 0.963 (0.961, 0.966) | <0.001 | 0.313 (0.275, 0.354) | <0.001 |
| Attention and calculation | 0.923 (0.917, 0.929) | <0.001 | 0.945 (0.939, 0.951) | <0.001 | 0.977 (0.975, 0.978) | <0.001 | 0.285 (0.256, 0.321) | <0.001 |
| Copy figure | 0.781 (0.754, 0.808) | <0.001 | 0.850 (0.822, 0.880) | <0.001 | 0.922 (0.914, 0.928) | <0.001 | 0.328 (0.279, 0.388) | <0.001 |
| Delayed-recall | 0.872 (0.861, 0.910) | <0.001 | 0.917 (0.912, 0.931) | <0.001 | 0.969 (0.967, 0.972) | <0.001 | 0.266 (0.238, 0.291) | <0.001 |
| Language | 0.926 (0.920, 0.932) | <0.001 | 0.950 (0.944, 0.956) | <0.001 | 0.975 (0.973, 0.977) | <0.001 | 0.320 (0.283, 0.365) | <0.001 |

† HR: hazard ratio; CI: confidence interval.

†Adjusted for age, sex, residence, smoking status, drinking status, tea drinking, regular physical activity, lifestyle, and eight kinds of self-reported disease
