# Supplementary material for: Mediation analysis of leisure activities on the association between cognitive function and mortality: a longitudinal study of 42,942 Chinese adults 65 years and older
Source: Epidemiol Health. 2022 Nov 27;44:e2022112. doi: 10.4178/epih.e2022112 (PMC10106552; doi:10.4178/epih.e2022112)
Supplement: Supplementary file 6 [file epih-44-e2022112-Supplementary-6.docx]

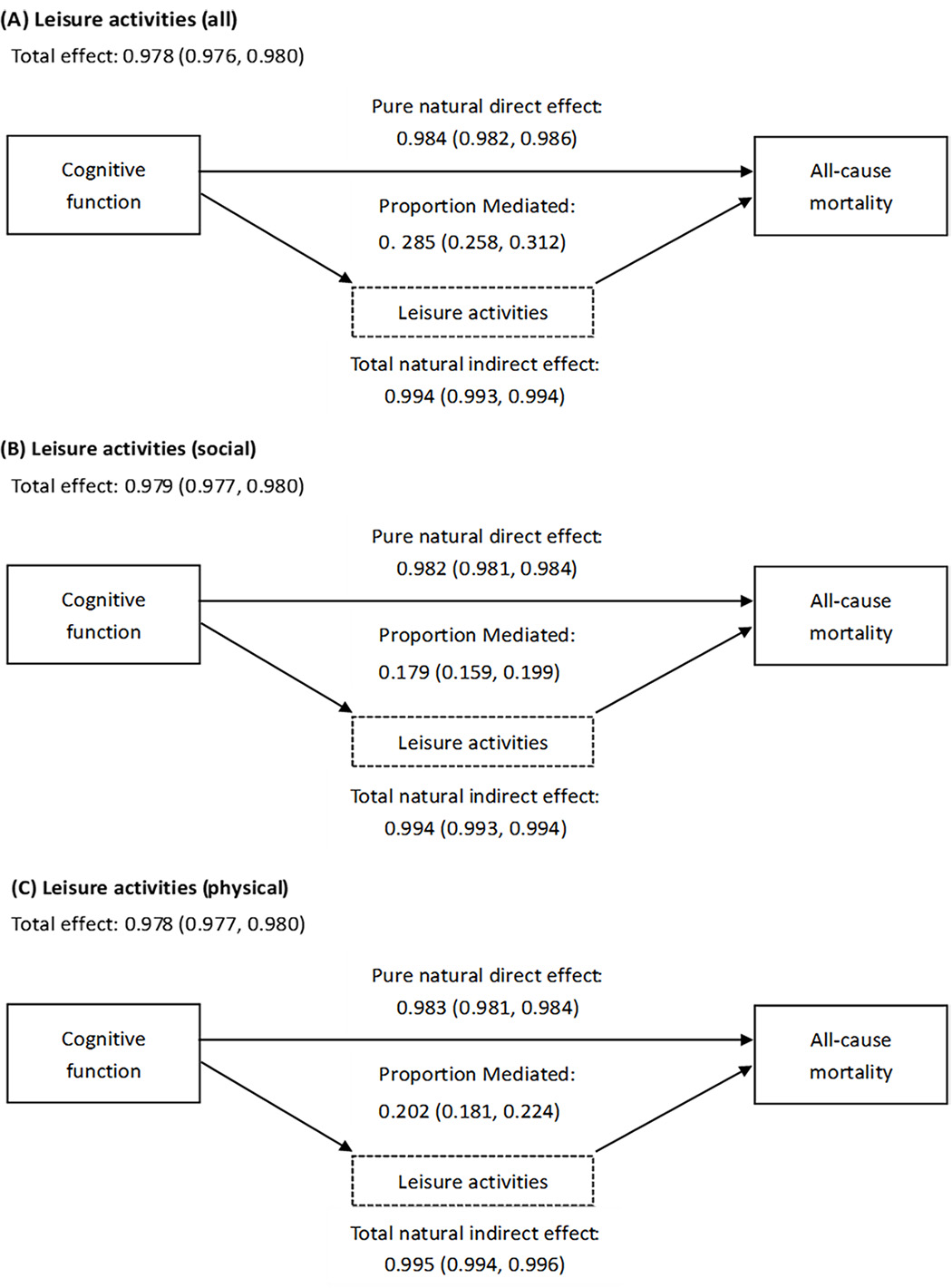


**Supplementary Material 6.** Sensitivity analyses of causal mediation of leisure activities on cognitive function and all-cause mortality by including participants who had missing values on key variables (adjusted for age, sex, residence, smoking status, drinking status, tea drinking, regular physical activity, lifestyle, and eight kinds of self-reported disease) (*n* = 42,942).
