# Supplementary material for: Mediation analysis of leisure activities on the association between cognitive function and mortality: a longitudinal study of 42,942 Chinese adults 65 years and older
Source: Epidemiol Health. 2022 Nov 27;44:e2022112. doi: 10.4178/epih.e2022112 (PMC10106552; doi:10.4178/epih.e2022112)
Supplement: Supplementary file 7 [file epih-44-e2022112-Supplementary-7.docx]

**Supplementary Material 7.** Sensitivity analyses for multivariable-adjusted hazard ratios and 95% confidence intervals of all-cause mortality by cognitive function and leisure activities among Chinese adults aged ≥65 years (exclude participants who died within two years of the follow-up) (*n* = 31,543)

|  | **Adjusted hazard ratio (95% confidence interval)** | ***P* value** |
| --- | --- | --- |
| **Cognitive function** |  |  |
| Not impaired | Re f. |  |
| Mild | 1.12 (1.07, 1.16) | <0.001 |
| Moderate | 1.26 (1.20, 1.33) | <0.001 |
| Severe | 1.37 (1.28, 1.47) | <0.001 |
| **Leisure activities** |  |  |
| Always | Re f. |  |
| Frequently | 1.26 (1.17, 1.36) | <0.001 |
| Sometimes | 1.46 (1.39, 1.54) | <0.001 |
| Rarely | 1.55 (1.48, 1.76) | <0.001 |

† Adjusted for age, sex, residence, smoking status, drinking status, tea drinking, regular physical activity, lifestyle, and eight kinds of self-reported disease.
